# Supplementary material for: “Head-to-Toe” Lipid Properties Govern the Binding and Cargo Transfer of High-Density Lipoprotein
Source: Membranes (Basel). 2024 Dec 6;14(12):261. doi: 10.3390/membranes14120261 (PMC11677176; doi:10.3390/membranes14120261)
Supplement: Supplementary file 1 [file membranes-14-00261-s001.zip › membranes-3324746-supplementary.pdf]

Figure 1

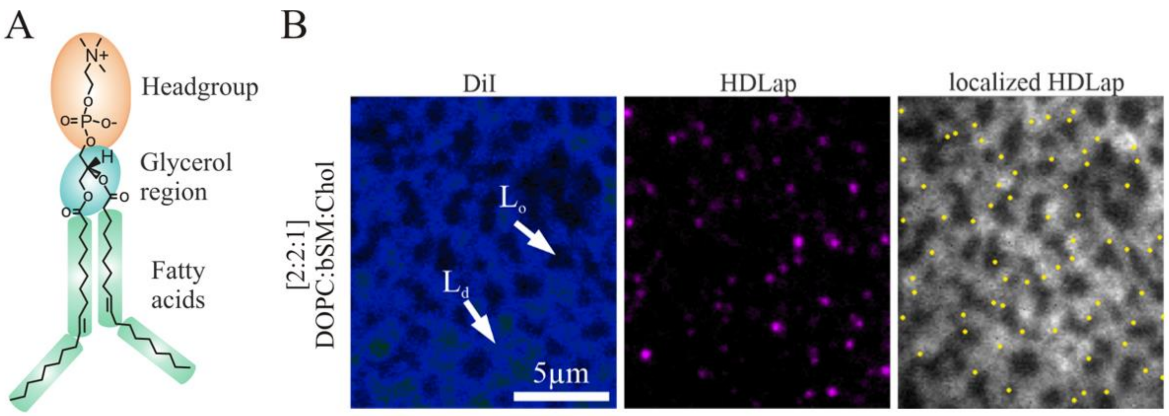

***Figure 1**: (**A**) Nomenclature used, exemplified by a structural model of a single DOPC (18:1) lipid. (**B**) HDL-associated protein (HDLap, fluorescently labeled with Atto 647) partitions preferentially to the $L_d$phase in PSLBs ([2:2:1] mixture of DOPC (18:1), bSM and Chol) supported on a glass surface. The bilayer was treated with DiI as an L_d phase marker (shown in the first color channel, corresponding to the image on the left) and HDL particles (shown in the second color channel, corresponding to the middle image, and represented as yellow dots in the right image, illustrating the center of mass.) (partition coefficient of K_p = 3 a.u. of HDLap (phase 1: L_d, phase 2: L_o)). The last image displays the positions of single HDLap-Atto 647 signals (indicated as yellow dots) overlaid with the DiI fluorescence image.*

Figure 2

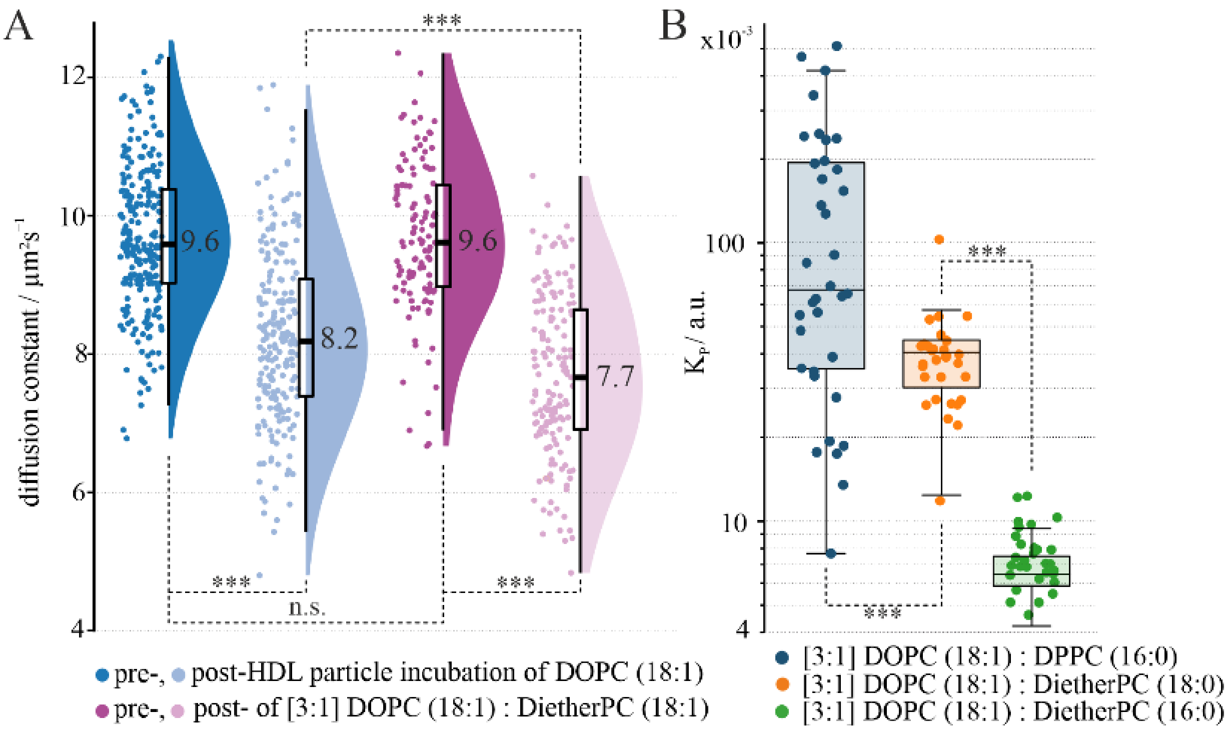

Figure 2**: HDL particle interaction with different lipid membranes. (**A**) shows the scatter-, box- and distribution- plot of the lateral diffusion constant of DPPE-ASR within a GUV membrane of DOPC (18:1) and DOPC (18:1) : DietherPC (18:1) [3:1] pre and post incubation with a HDL particle solution. Each individual point depicts the diffusion constant obtained from one measurement on the top GUV membrane: pre HDL incubation (dark blue) ($D_{DOPC\ (18:1)\ preHDL}$ =9.6 µm²/s ± 0.1 µm²/s, N=237) and (dark magenta) ($D_{DOPC\ (18:1)\ :\ Diether\ PC\ (18:1)\ preHDL}$ =9.6 µm²/s ± 0.1 µm²/s. N=114), post HDL incubation (light blue) ($D_{DOPC\ (18:1)\ postHDL}$ =8.2 µm²/s ± 0.1 µm²/, N=208) and (light magenta) ($D_{DOPC\ (18:1)\ :\ Diether\ PC\ (18:1)\ postHDL}$ =7.7 µm²/s ± 0.1 µm²/s, N=153)(n.s. not significant, *** p<0.001@ α=5%) (**B**) shows the boxplot chart of the calculated partitioning coefficient K_p (phase 1: L_d phase (i.e., DOPC); phase 2: gel phase (i.e., DPPC, DietherPC)) regarding HDLap localization in respect of three different (i.e., lipid composition) PSLBs. (Median ± SE) DOPC (18:1) : DPPC (16:0) [3:1] (blue): K_p = 68 x 10^{-3} ± 22 x 10^{-3} a.u. (N= 36); DOPC (18:1) : Diether PC (18:0) [3:1] (orange): K_p = 40 x 10^{-3} ± 3 x 10^{-3} a.u. (N= 30); DOPC (18:1) : Diether PC (16:0) [3:1] (green): K_p = 6 x 10^{-3} ± 3 x 10^{-4} a.u. (N= 30) (p<0.001 @ α=5%).*

Figure 3

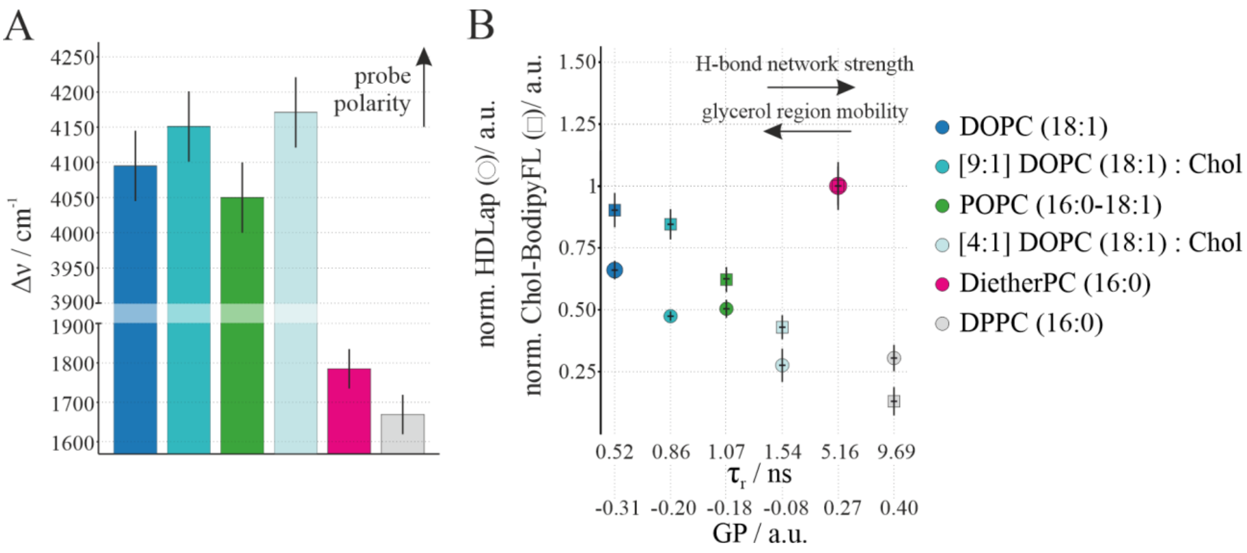

***Figure 3**: HDL particle interaction with lipid membranes of different probe polarity/GP value/ hydrogen-bond network strength. (**A**) The bar chart depicts the total amount of fluorescence shift Δv of the Laurdan emission for different GUV membrane compositions. Intrinsic uncertainty for Δv parameter is 50 cm^{-1}. The Δv parameter is directly related to the hydrogen-bond network level of sn-1 carbonyls. Higher magnitude of fluorescence shift reflects a higher probe polarity. (**B**) shows the relaxation time τ_r / GP value for different GUV membranes (Supplemental Table 1) in relation to normalized (i.e., both DietherPC (16:0) values were set to 1) HDL-associated protein (HDLap) Atto 647 (○) and normalized Chol-BodipyFL (□) signal per pixel (Supplemental Figure 4). Higher magnitude of GP value and relaxation time τ_r reflect a lower glycerol region mobility and stronger hydrogen-bond network, respectively. Error bars indicate standard error of the mean.*

Figure S1

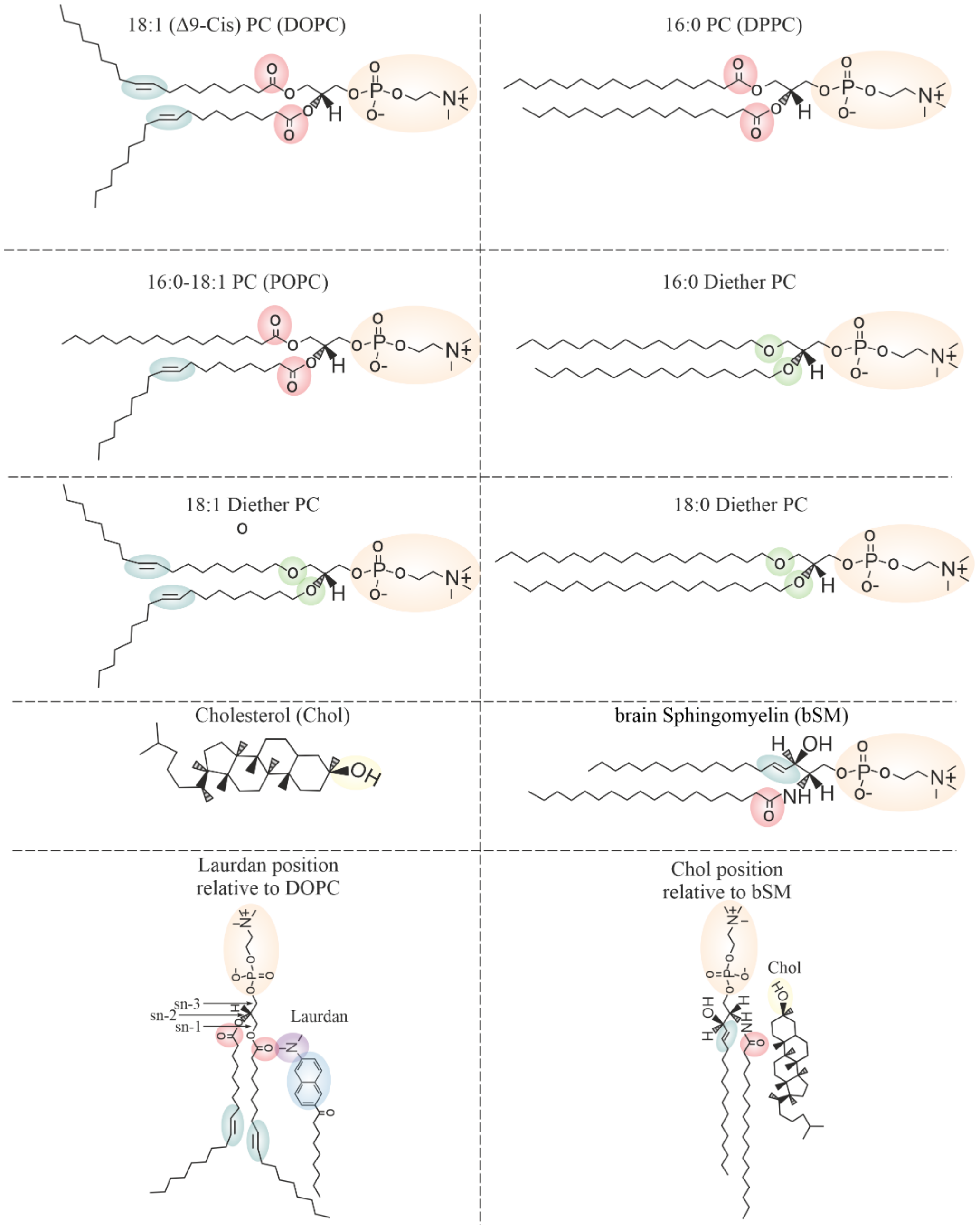

Supplement Figure 1*: Overview of the structure of the PC lipids used: Ester-linked: DOPC (18:1), DPPC (16:0), POPC (16:0-18:1) ; Ether-linked: Diether PC (16:0), Diether PC (18:1), Diether PC (18:0); Chol, bSM. The last row shows a schematic representation of a DOPC (18:1) lipid (stereospecific nomenclature (sn) of the glycerol backbone C-atoms) or bSM, respectively and the position of Laurdan or Chol. SM interacts with the Chol's 3-OH group via its own phosphate oxygen and its NH group takes part in intermolecular hydrogen-bonds among bSM molecules* [1,2].

Figure S2

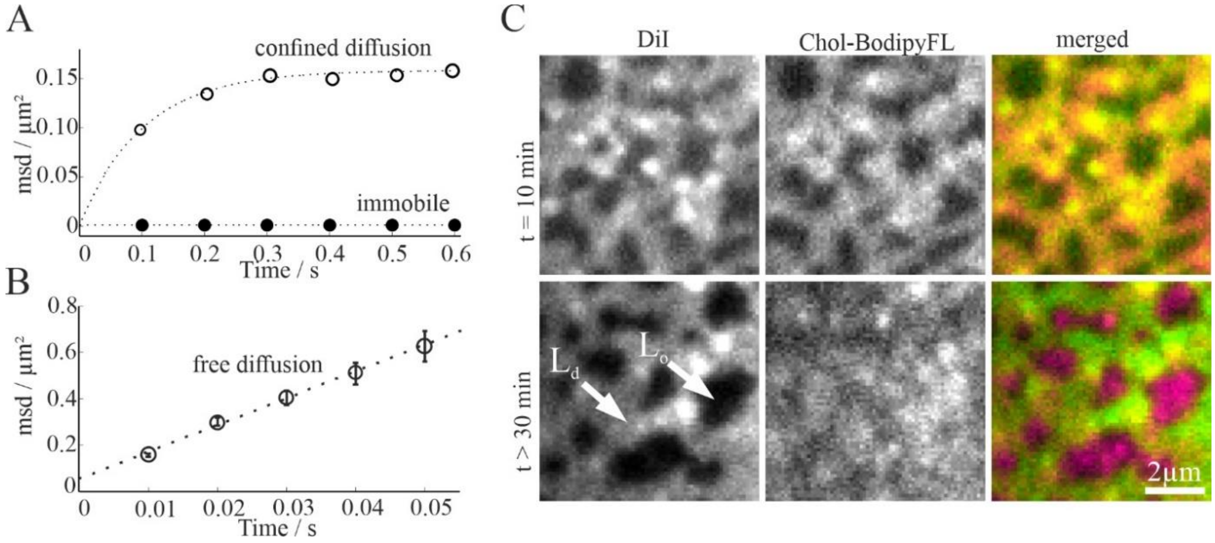

***Supplement Figure 2**: Lipid bilayers were prepared from a [2:2:1] mixture of DOPC (18:1), bSM and Chol and formed on glass surfaces. Panels (**A**) and (**B**) show the results of a tracking analysis of the HDL-associated protein–Atto 647 signal (**A**), and of Chol-BodipyFL signal (**B**). The mean square displacement (msd) is plotted as function of the time-lag. For the protein signal, two fractions of different mobility were identified: a mobile one (31%; open circles) was fitted with Methods Eq. 2 for confined diffusion, yielding a domain radius R = 400 nm and a mobility D = 0.38 $\mu m^2/s$; R can be interpreted as the mean free path length of the particles within the L_d phase. The remaining fraction (69%; full circles) showed no detectable mobility (D = $3.5 \cdot 10^{-5} \mu m^2/s$). Chol-BodipyFL showed free diffusion with D = 2.75 ± 0.07 $\mu m^2/s$ (Methods Eq. 1). (**C**) Representative images of PSLBs after incubation for 10 minutes (upper row) and for more than 30 minutes (lower row). DiI was used as marker for the L_d phase. The Chol-BodipyFL released via HDL particles showed in the first 10 minutes a clear preference for the L_d phase and switch after >30 minutes into the L_o phase.*

Figure S3

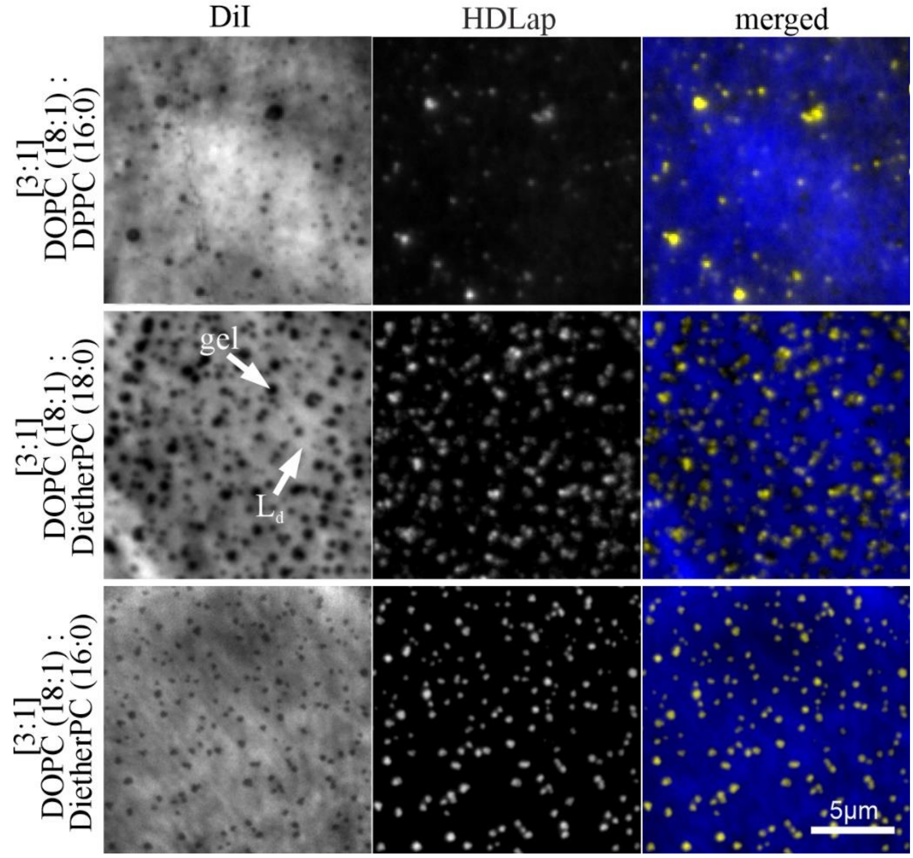

***Supplement Figure 3**: Partitioning of HDL-associated protein (HDLap) on PSLBs with different lipid compositions: DOPC (18:1) : DPPC (16:0) [3:1] (top row); DOPC (18:1) : Diether PC (18:0) [3:1] (middle row); DOPC (18:1) : Diether PC (16:0) [3:1] (bottom row). The images (obtained in TIRF configuration) represent the DiI signal within the L_d phase (first column), the HDLap Atto 647 signal (second column) and both merged channels (third column).*

Figure S4

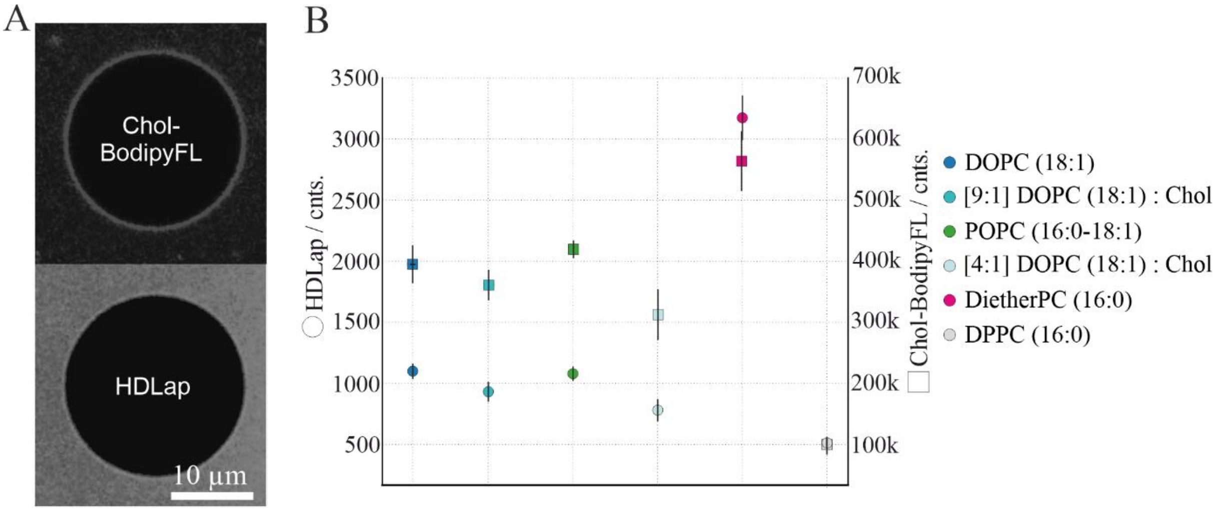

Supplement Figure 4: *HDL particle interaction with lipid membranes. (A) Representative images (left) of a single GUV in the Chol-BodipyFL channel and the HDLap-Alexa Fluor 647 channel are shown. (B) The right panel shows the Chol-BodipyFL versus the HDLap-Alexa Fluor 647 signal per pixel for single vesicles. Vesicles made of POPC (16:0-18:1), DOPC (18:1), [9:1] DOPC (18:1) : Chol, and [4:1] DOPC (18:1) : Chol, Diether PC (18:0) and DPPC (16:0) were studied. Circles indicate HDLap-Alexa Fluor 647 channel, squares indicate Chol-BodipyFL channel. Averaged Chol-BodipyFL and HDLap-Alexa Fluor 647 signal are obtained from GUVs made from the indicated lipid composition. Error bars indicate the standard error of the mean.*

Table S1:

	T_m / °C	Δv / cm^{-1}	GP value / a.u.	τ_r / ns
DOPC (18:1)	-17 [3]	4095±50	-0.310±0.005	0.52
[9:1] DOPC (18:1) : Chol		4151±50	-0.200±0.004	0.86
POPC (16:0-18:1)	-3 [4]	4050±50	-0.177±0.004	1.07
[4:1] DOPC (18:1) : Chol		4171±50	-0.078±0.002	1.54
DietherPC (16:0)	44 [5]	*1785±50**	0.272±0.001	5.16
DPPC (16:0)	41 [6]	*1669±50**	0.395±0.001	9.69

***Supplement Table 1**: Data of the different GUV membranes presented in Figure 3: the phase transition temperature (i.e., "melting" temperature T_m), and the measured probe (i.e., Laurdan) parameters: the fluorescence shift Δv, the Generalized Polarization (GP) value, and the relaxation time τ_r. (* Δv parameters are ill estimated because the fluorescence lifetime of Laurdan is significantly faster than the relaxation process. Therefore, the final solvent relaxed state cannot be probed and $v(\infty)$ is an empirical parameter).*

References

1. Bittman, R.; Kasireddy, C.R.; Mattjus, P.; Slotte, J.P. Interaction of Cholesterol with Sphingomyelin in Monolayers and Vesicles. *Biochemistry* **1994**, *33*, 11776–11781, doi:10.1021/bi00205a013.

2. Bhattacharya, S.; Haldar, S. Interactions between Cholesterol and Lipids in Bilayer Membranes. Role of Lipid Headgroup and Hydrocarbon Chain–Backbone Linkage. *Biochimica et Biophysica Acta (BBA) - Biomembranes* **2000**, *1467*, 39–53, doi:10.1016/S0005-2736(00)00196-6.

3. Ulrich, A.S.; Sami, M.; Watts, A. Hydration of DOPC Bilayers by Differential Scanning Calorimetry. *Biochimica et Biophysica Acta (BBA) - Biomembranes* **1994**, *1191*, 225–230, doi:10.1016/0005-2736(94)90253-4.

4. Koster, K.L.; Webb, M.S.; Bryant, G.; Lynch, D. V. Interactions between Soluble Sugars and POPC (1-Palmitoyl-2-Oleoylphosphatidylcholine) during Dehydration: Vitrification of Sugars Alters the Phase Behavior of the Phospholipid. *Biochimica et Biophysica Acta (BBA) - Biomembranes* **1994**, *1193*, 143–150, doi:10.1016/0005-2736(94)90343-3.

5. Matsuki, H.; Miyazaki, E.; Sakano, F.; Tamai, N.; Kaneshina, S. Thermotropic and Barotropic Phase Transitions in Bilayer Membranes of Ether-Linked Phospholipids with Varying Alkyl Chain Lengths. *Biochimica et Biophysica Acta (BBA) - Biomembranes* **2007**, *1768*, 479–489, doi:10.1016/j.bbamem.2006.10.005.

6. Chiu, M.; Prenner, E. Differential Scanning Calorimetry: An Invaluable Tool for a Detailed Thermodynamic Characterization of Macromolecules and Their Interactions. *J Pharm Bioallied Sci* **2011**, *3*, 39, doi:10.4103/0975-7406.76463.
